# Oxidative stress and autophagy-related changes during retinal degeneration and development

**DOI:** 10.1038/s41419-018-0855-8

**Published:** 2018-07-24

**Authors:** Laura Trachsel-Moncho, Soledad Benlloch-Navarro, Ángel Fernández-Carbonell, Dolores Tania Ramírez-Lamelas, Teresa Olivar, Dolores Silvestre, Enric Poch, María Miranda

**Affiliations:** 1 Departamento Ciencias Biomédicas, Facultad de Ciencias de la Salud, Universidad Cardenal Herrera-CEU, CEU Universities, Valencia, Spain; 2 Departamento Farmacia, Facultad de Ciencias de la Salud, Universidad Cardenal Herrera-CEU, CEU Universities, Valencia, Spain

## Abstract

Retinitis pigmentosa (RP) is an inherited retinopathy that leads to photoreceptor loss. RP has been related to oxidative stress, autophagy, and inflammation. This study aimed to identify changes in the levels of oxidative stress and autophagy markers in the retina of control and rd10 mice during different phases of retinal development. Changes in the retinal oxidation system were investigated by measuring the levels of oxidized and reduced glutathione (GSH/GSSG), retinal avidin-positive cells, and 4-hydroxynonenal (4-HNE) staining intensity. Autophagy characterization was explored by measuring the levels of microtubule-associated protein 1 light chain 3 (LC3), beclin, autophagy-related proteins 5 and 7 (Atg5 and Atg7), and lysosomal associated membrane protein-2A (LAMP-2A). At P28 retinal GSH concentrations decreased in rd10 mice compared to the controls. No differences were found in retinal GSSG concentrations between the control and rd10 mice. There was an increase in retinal GSSG concentrations and a decrease in the GSH/GSSG ratio in the control and rd10 mice at P21 and P28 compared to P13. We observed an increase in avidin-positive cells in rd10 retinas. 4-HNE was increased in rd10 retinas at P13, and it also increased in control mice with age. We did not observe any differences in the retinal levels of LC3II/I ratio, Beclin, Atg5, or Atg7 in the rd10 mice compared to the controls. There was an increase in the LAMP-2A concentrations in the control and rd10 mice with development age (P28 concentrations vs. P13). Although only slight differences were found in the oxidative stress and autophagy markers between the control and rd10 mice, there were increases in the GSSG, 4-HNE, and LAMP-2A with age. This increase in the oxidative stress and chaperone-mediated autophagy has not been described before and occurred just after the mice opened their eyes, potentially indicating a retinal response to light exposure.

## Introduction

Retinitis pigmentosa (RP) belongs to a clinically and genetically heterogeneous group of retinopathies. It is caused by genetic mutations that lead to a progressive loss of retinal photoreceptors and the alteration of other retinal layers^1^.

RP has a low prognostic cure rate^1^. The main visual symptoms include a progressive decrease in visual acuity that predominantly affects night vision and the peripheral field^2,3^. The RP genetic defects first affect the rods, inducing their degeneration. Once the rods have degenerated, the cones also die, resulting in blindness.

Oxidative stress is known to play a key role in the RP pathogenesis. Several hypotheses have been put forth for the cone degeneration that follows rod death in RP. One hypothesis attributes the cone degeneration to oxidative stress, and it is explained by the markedly elevated oxygen levels within the retina after rod death^4^. Antioxidants have been shown to diminish cone death in several RP animal models^5–7^.

Rods are highly metabolically active cells with an elevated oxygen consumption rate^8^. During RP progression, retinal oxygen consumption diminishes^8^. Although the oxygen demand is significantly low, the blood supply remains the same^9^, leading to a hyper-oxidation state that causes oxidative damage. This oxidative damage affects all the cells in the retina, including the cones, which ultimately die by apoptosis^10^, and exacerbates the death of the rods. The accumulation of O_2_^−^ will lead to lipid, protein, and DNA damage^8,11^. This process is known as the oxygen toxicity hypothesis.

Oxidative stress markers have been found to be altered in RP patients^12^. Our previous results have shown that there is a negative correlation between retinal glutathione (GSH) concentration and the number of terminal deoxynucleotidyl transferase-mediated UTP nick end labeling (TUNEL) positive cells in RP mice that have received an oral antioxidation combination of zeaxanthin, lutein, α-lipoic acid, glutathione, and Lycium barbarum extract^13^. GSH serves as the cell’s first line of defense against oxidative stress and has several important functions. GSH transports and stores cysteine, assists in the detoxification of xenobiotics, and is a cofactor in isomerization reactions^14,15^. GSH has also been suggested to play a role in apoptosis regulation^16–19^.

Oxidative stress and thiol redox metabolism are also related to autophagy. Lysosomes are susceptible to oxidative stress and membrane destabilization, leading to lysosomal membrane permeabilization, a key factor in the autophagy process^20,21^. Autophagy is a highly conserved evolutionary process in eukaryotic cells. It allows cells to control the replacement of long-lived proteins and cytoplasmic organelles, degrading them via lysosomes^22^. This process takes place on a small scale at baseline levels in virtually all cells, playing an important role in homeostatic functions. However, it is induced as an adaptive catabolic process in response to stress caused by starvation, growth factor deficiencies, hypoxia, or protein aggregate accumulation^23^. Three types of autophagy have been described in mammals: macroautophagy, microautophagy, and chaperone-mediated autophagy (CMA)^24^. All three autophagy types result in lysosomal degradation through different mechanisms^23^.

Autophagy is generally considered to be a cytoprotective mechanism, maintaining homeostasis under starvation conditions and eliminating defective proteins, damaged organelles, and disease-causing pathogens. However, autophagy activation may also be harmful, either by allowing some cancer cells to become resistant to certain chemotherapeutics or by leading to undesirable cell death^22,25^. Defective autophagy has been associated with the pathogenesis of various diseases, such as cancer and certain types of neuronal degenerations, as well as the aging process.

It has been suggested that the rod death in various RP animal models is carried out through apoptosis and that cone death is a secondary response to rod death^10,26,27^. Although the main photoreceptor death pathway could be apoptosis, other types of cell degeneration may also be involved, such as autophagy. Several studies have shown that autophagy is involved in the survival of the rods and cones in mice^28,29^; however, little is known about the role of autophagy in the survival or death of the photoreceptor cells in RP or in the pathophysiology of other retinal diseases.

Oxidative stress and autophagy have also been related to retinal development. Two phases of programmed cell death have been described during retinal development^30^. An early phase takes place at the same time as neurogenesis, cell migration, and differentiation. Later, cell death that mostly affects neurons occurs when connections and synapses are formed. This phase primarily takes place during the first two postnatal weeks and is almost completed by the end of the third week^30^. Ganglion cell degeneration is noticeable during the first 11 days after birth; amacrine cells die between postnatal (P) days 3–8 (P3–P8) days; bipolar and Muller cell death reaches a peak at P8–P11^30^. The formation of the outer plexiform layer on P5 divides the rods into two groups. Rod death persists during the successive two weeks until P18, after which cell death within the retina is circumscribed to occasional rod degeneration, which is completed by P30^30^.

Autophagy genes are required for the phagocytic removal of corpses during the programmed cell death that occurs during mouse retinal development^31^. Autophagy effectively removes apoptotic cells, preventing damaging inflammatory responses during development^32^. In addition, autophagy eliminates oxidatively damaged molecules. The retina is highly susceptible to oxidative stress as it has a large amount of polyunsaturated fatty acids, it consumes large quantities of oxygen, and it is exposed to visible light. In mice, light exposure begins when the eyes open at around P11–P13, a period when the development and maturation of the retina have not yet finished.

This work aimed to evaluate the retinal changes in oxidative stress and autophagy markers in control and rd10 mice, an RP animal model, through different phases of retinal development.

## Results

### Characterization of the cell death timeline during retinal development in control and rd10 mice

In the rd10 mouse model, retinal degeneration starts 18 days after birth, and morphological changes can be detected after 20 days^33^. Some authors have described alterations to this degeneration pattern that are likely due to different housing light intensities^34,35^, so we characterized the rd10 photoreceptor degeneration timeline specific to our housing conditions.

**Fig. 1:**
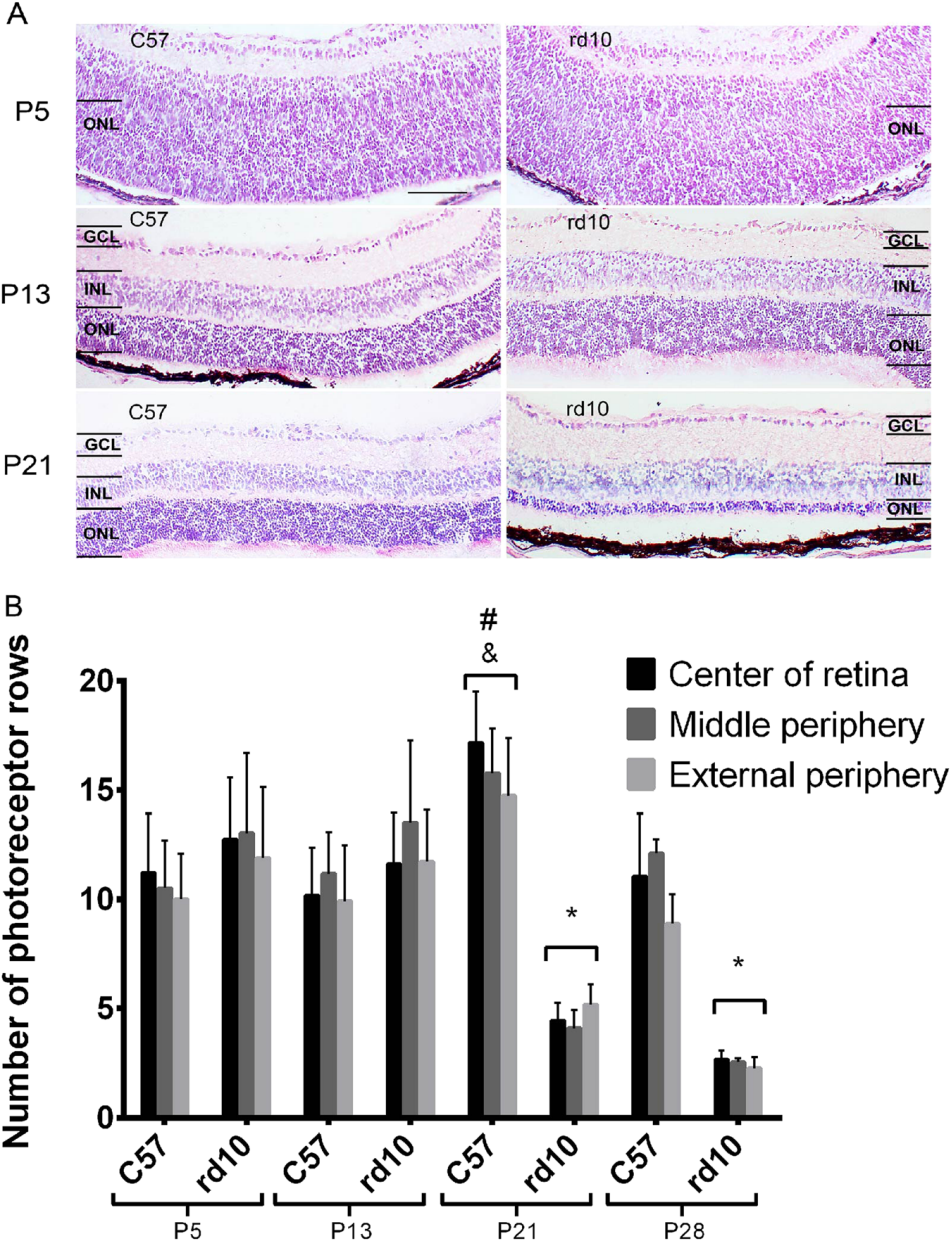
The number of photoreceptor rows in different areas of the retina. **a** An illustration of the degradation of the retina at P5, P13, and P21 for the C57 (control) on the left, and the rd10 mice on the right. **b** The mean number of photoreceptor rows in the control and rd10 mice; error bars indicate the standard deviation (*p < 0.03 vs every other group; ^#^*p* < 0.05 vs P13 rd10; ^&^*p* < 0.05 vs P5 C57 and P13 C57)

To evaluate the photoreceptor cell loss in rd10 mice, we measured the number of rows of cells at the outer nuclear layer (ONL) in different retinal areas (external periphery, middle periphery, and center of the retina) at P5, P13, P21, and P28 in C57 (control) and rd10 mice (Fig. 1a, b). At P5 the retina was not completely developed (Fig. 1a). In addition, a very relevant photoreceptor cell loss was observed in the rd10 mice at P21 and P28, compared to all other ages (Fig. 1b).

**Fig. 2:**
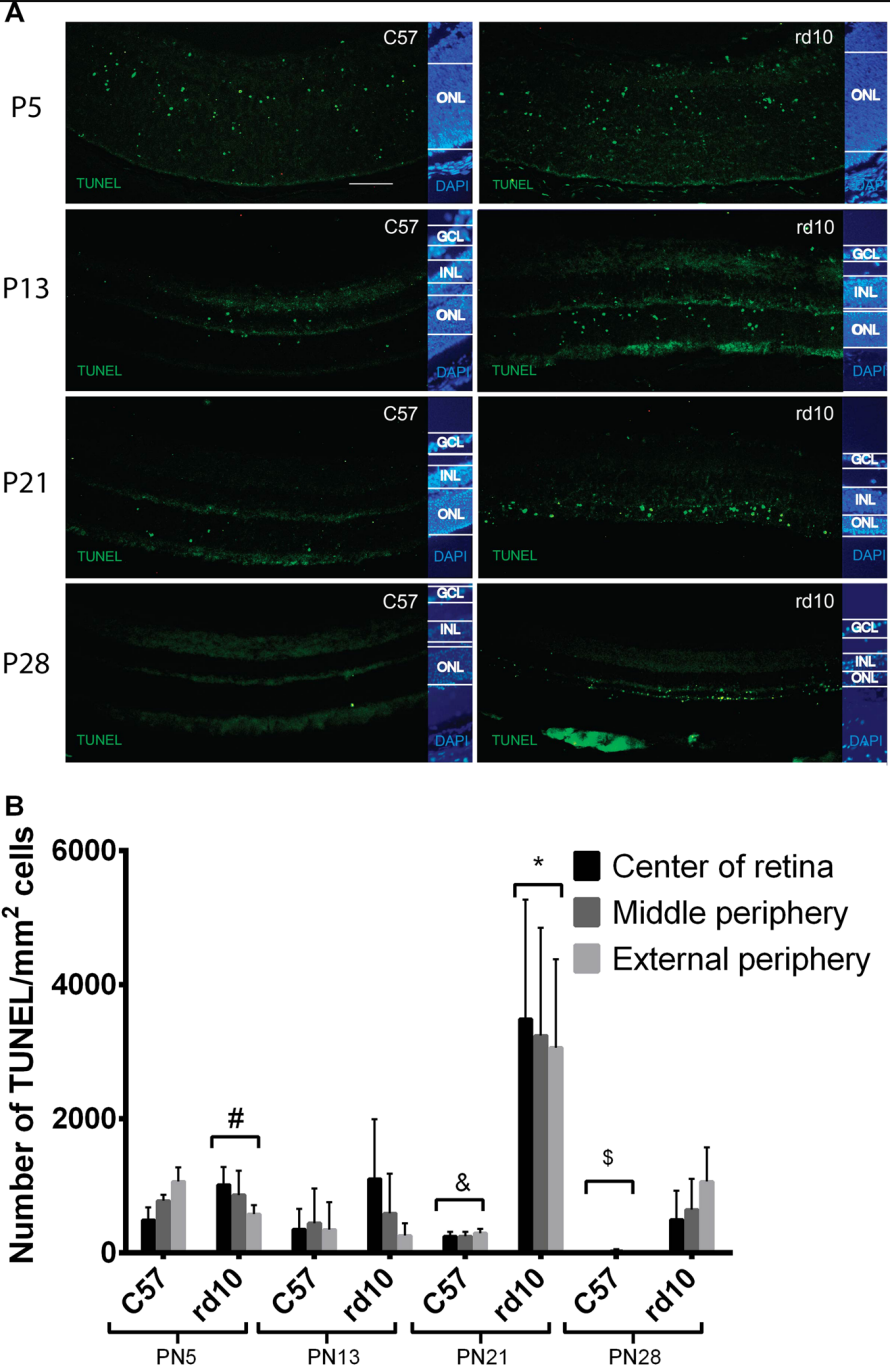
Retinal cell death. **a** Representative images of TUNEL staining in green, accompanied by DAPI staining in blue. The GCL, INL and ONL layers are indicated. The picture in the leftmost group is the control mice, while the rightmost is the rd10 mice (*p < 0.03 vs every group; ^#^*p* < 0.05 vs P13 C57 and P21 C57; ^&^*p* < 0.05 vs P5 C57; ^$^*p* < 0.05 vs P21 rd10 and P28 rd10). **b** The mean number of TUNEL positive cells in the control and rd10 mice retina, error bars indicate the standard deviation. (^#^*p* < 0.03 vs every other group; ^#^*p* < 0.05 vs P13 rd10; ^&^*p* < 0.05 vs P5 C57 y P13 C57).

TUNEL assay was also accomplished (Fig. 2a, b). At P5, the TUNEL-labeled cells were observed in most retinal layers in both C57 and rd10 mice (Fig. 2a). In the rd10 retinas we observed that the number of dying cells in the ONL particularly increased at P21 compared to control retina. At P28, retinal cell death diminished slightly in the rd10 mice (Fig. 2b).

### Characterization of the thiol metabolism and glutamate content in the rd10 mice and during retinal development

Reduction in GSH concentrations and disproportionate GSH/GSSG ratios have been associated with several neuropathological processes, including RP, Parkinson´s disease, diabetes, and Alzheimer´s disease^13,36–38^. There is evidence that the conservation of cellular GSH defends against oxidant-induced apoptosis^39^.

We selected P13, P21, and P28 to measure the retinal concentrations of GSH, reduced glutathione (GSSG) (by an HPLC method) and the GSH/GSSG ratios. These time points correspond to the early, intermediate (around the peak of rod cell death), and final phases of the genetic retinal degeneration. At these postnatal days the outer nuclear and plexiform layer is formed and easily differentiated. At P28, most of the cells remaining in the retina of the rd10 mice are cones, but they do not have a normal morphology, so we can consider P28 as the initial stage of cone mutation-independent degeneration.

**Fig. 3:**
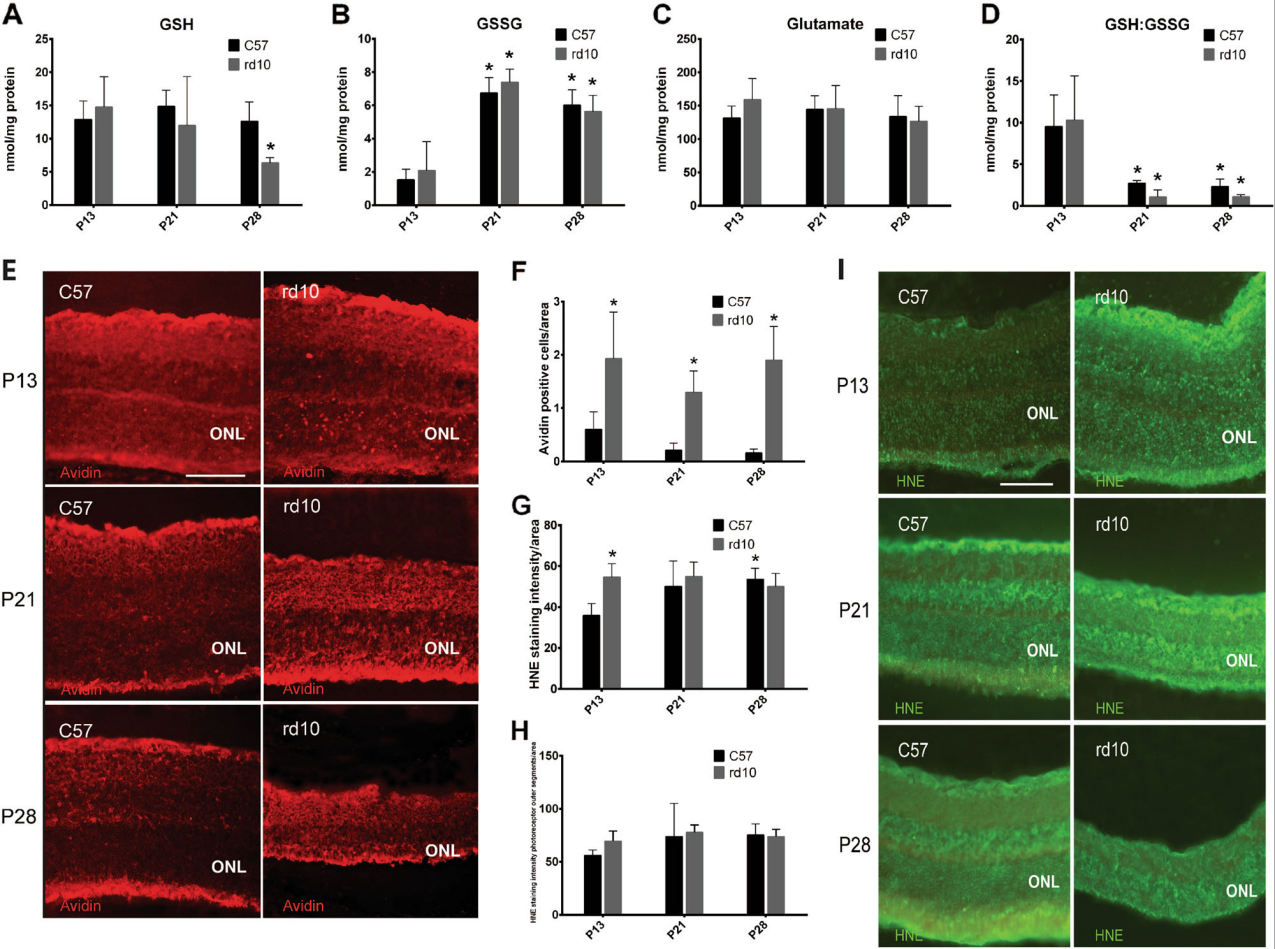
Oxidative stress markers in control and rd10 retinas. **a** Mean retinal levels of GSH at different ages. **b** Mean retinal levels of GSSG at different ages. **c** Mean retinal levels of glutamate at different ages. **d** The retinal GSH:GSSG ratio in the retina at different ages (*p < 0.01 vs P13). **e** Representative images of the avidin staining in red in different retinal sections. **f** Mean avidin-positive cell number in the control and rd10 different mice types, where error bars indicate the standard deviation (*p < 0.03 vs. each control). **g** Mean 4-HNE retinal intensity staining in the control and rd10 different mice types, where error bars indicate the standard deviation (*p < 0.05 vs. C57 P13). **h** Mean 4-HNE intensity staining in the photoreceptor outer segments in the control and rd10 different mice types, where error bars indicate the standard deviation. **i** Representative images of the 4-HNE staining in green in different retinal sections

Figure 3a shows the retinal GSH concentrations in control and rd10 mice at P13, P21, and P28. There were no statistically significant differences in the retinal GSH concentration of control and rd10 mice at P13 and P21. However, we detected a significant reduction in the retinal GSH concentration in the rd10 mice when compared to the control mice at P28 (*p* < 0.01). No statistically significant differences were observed in the retinal GSSG concentrations between control and rd10 retinas at any of the studied ages (Fig. 3b). Interestingly, there was an increase in retinal GSSG concentrations (Fig. 3b) and a decrease in the GSH/GSSG ratios (Fig. 3d) in both control and rd10 mice at P21 and P28 compared with P13 (just after eyes mice opening exposing the retina to light). Finally, no significant differences were observed in retinal glutamate (the major excitatory neurotransmitter in the retina) concentrations in control and rd10 mice at any of the studied ages (Fig. 3c).

We sacrificed some mice after 4 h of darkness and determined their retinal GSH and GSSG concentrations as well as the GSH/GSSG ratios to know if their concentrations and possible variations could be subjected to diurnal variation with lighting conditions. Our results demonstrate that during night GSH and GSSG did not change and were similar to the values obtained during the day (Supplementary Figure 1).

### Changes in other markers of oxidative stress markers

To better explain changes in oxidative stress with age and in the rd10 retinas, avidin and 4-HNE were also measured. Avidin can be used to identify oxidatively damaged DNA as previous studies have demonstrated that staining of retinas with 8-oxoguanine antibody and avidin resulted in co-labeling of cells in the ONL^6^. This has been explained because the structure of 8-oxoguanine is similar to the conventional ligand for avidin (biotin)^6^. 4-HNE is a toxic product of lipid oxidation. Our results demonstrate that avidin did not increase with age, but that avidin-positive cells were increased in rd10 retinas compared to controls (Fig. 3e, f). Regarding lipid peroxidation, our results show 4-HNE staining in all retinal layers, and a particularly intense staining in the outer segments of the photoreceptors (Fig. 3i). We quantified the intensity of this staining in the entire retina, but also in the photoreceptor outer segments (Fig. 3g, h). No changes were observed in the 4-HNE staining in the photoreceptor outer segments. However, an increase in the intensity of the staining was observed in the control retinas after mice opened their eyes (at P21 and P28). In rd10 retinas the intensity of 4-HNE was increased compared to control retinas at P13.

### Changes in the expression of autophagy markers in the control and rd10 mice during retinal development

Since the rd10 mice showed a marked cell death during retinal development, we analyzed whether autophagy was linked to this process, and how macroautophagy and CMA changed during the disease course and retinal development. We analyzed the protein levels of the following macroautophagy markers: microtubule-associated protein 1 light chain 3 (LC3); Beclin and the autophagy-related proteins 5 and 7 (Atg5 and Atg7). We also monitored a CMA marker: the lysosomal associated membrane protein-2A (LAMP-2A). The retinal concentrations of all these proteins were determined by western blot at P13, P21, and P28.

**Fig. 4:**
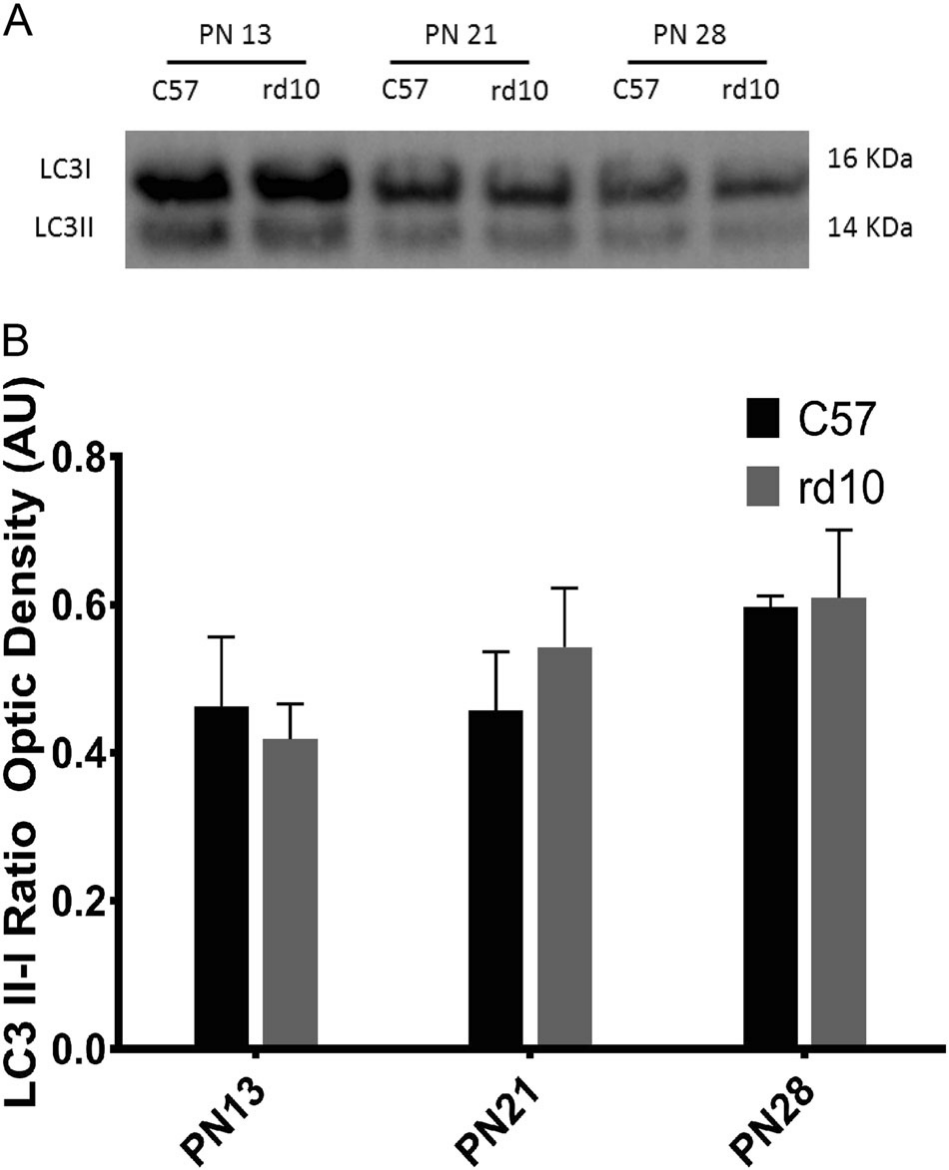
Detection of the LC3 protein by western blot in the different age groups. **a** Image of the western-blot bands for both the control and rd10 at different ages. **b** The graph represents the optical density quantification of the bands (ratio LC3 II-I) for each experimental group

**Fig. 5:**
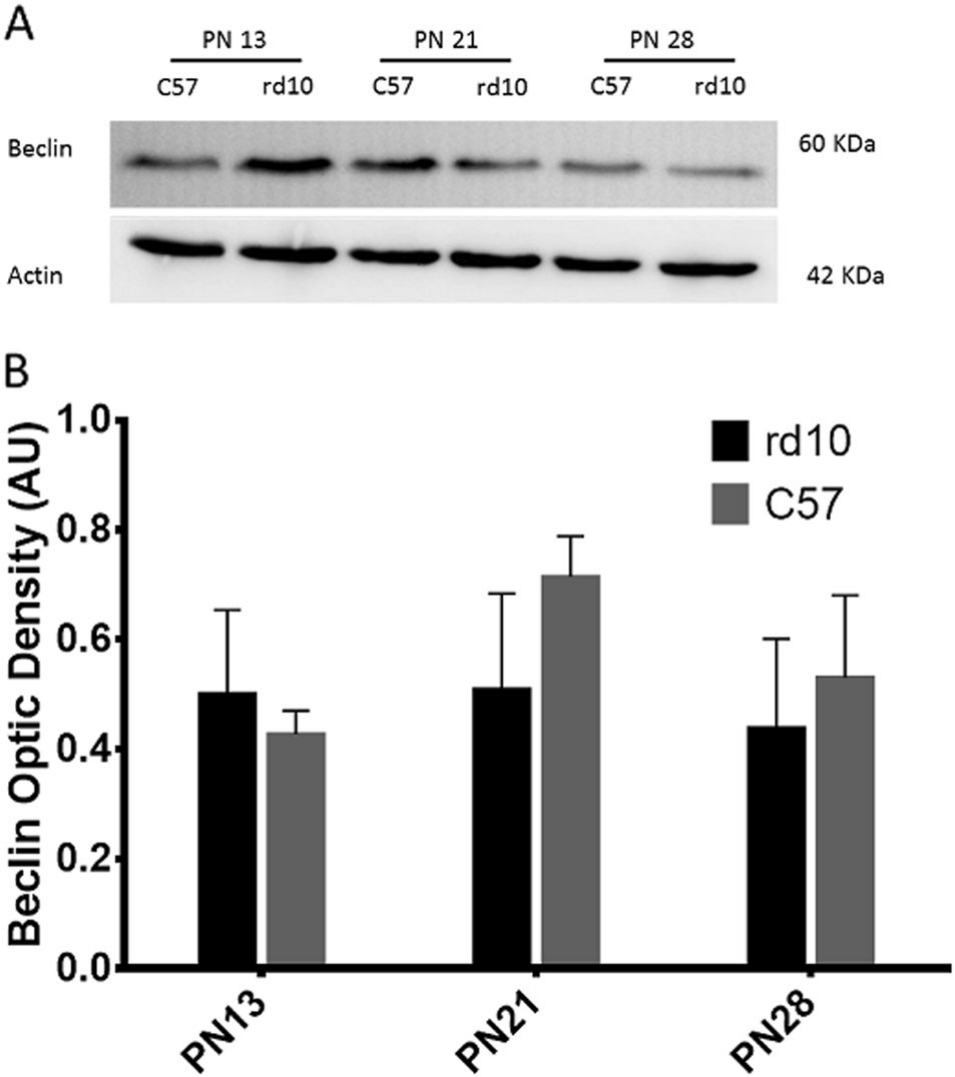
Detection of the Beclin protein by western blot in the different age groups. **a** Image of the western-blot bands for both the control and rd10 mice at different ages. **b** The graph represents the optical density quantification of the bands for each experimental group

The LC3 II-I ratio is one of the most common markers studied in autophagy, while Beclin is considered a marker for the initiation of autophagosome formation^42^. As shown in Figs. 4 and 5, LC3 II-I ratio and the Beclin’ score were normally distributed for all group combinations of strain and age, and there was no significant statistical interaction between the strain and the ages. We did not find any statistically significant differences between the LC3 and Beclin levels between control and rd10 mice, and no differences were observed in the expression of these two proteins independent of the retinal developmental age.

**Fig. 6:**
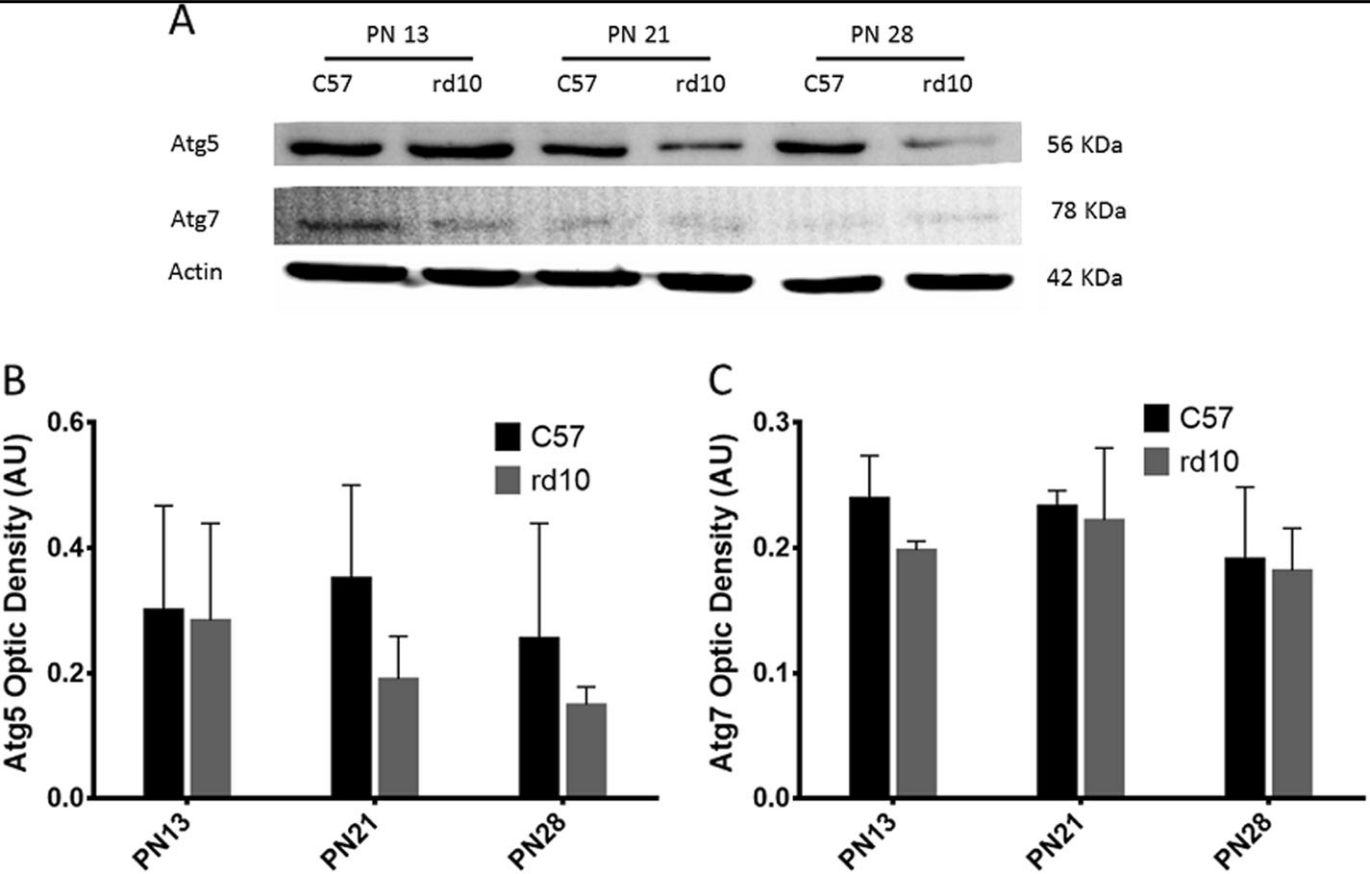
Detection of the Atg5 and Atg7 proteins by western blot in the different age groups. **a** Image of the western-blot bands for both the control and rd10 mice at different ages. **b** The graph represents the optical density quantification of the Atg5 bands for each experimental group. **c** The graph represents the optical density quantification of the Atg7 bands for each experimental group

The levels of Atg5 and Atg7, both implicated in autophagosome elongation, were also measured in the retinas of both control and rd10 mice. Our results showed a tendency to decrease in Atg5 protein levels during the postnatal development of both control and rd10 mice, though this decrease was not significant. Retinal samples from rd10 mice showed a greater decrease in the levels of Atg5 levels at P21 and P28 compared to controls, which coincides with the rod apoptosis peak (Fig. 6).

**Fig. 7:**
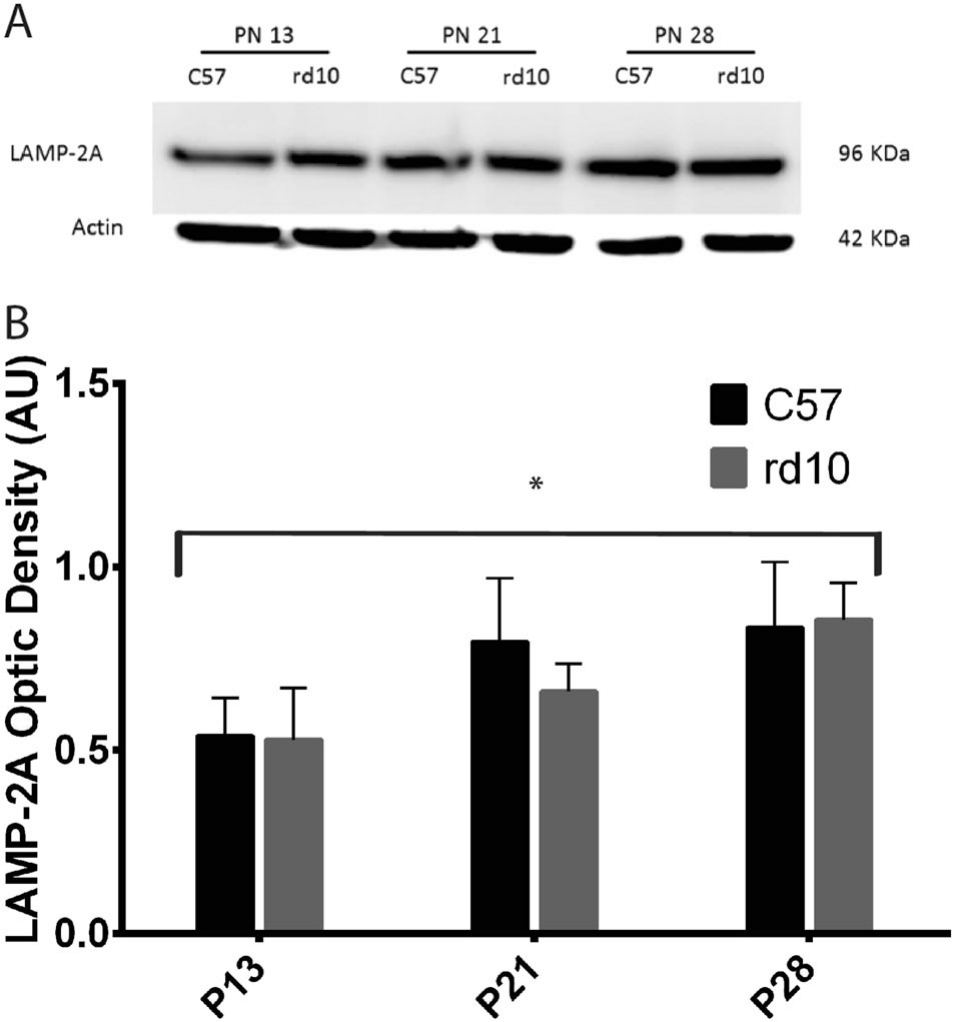
Detection of the LAMP-2A protein by western blot in the different age groups. **a** Image of the western-blot bands for both the control and rd10 mice at different ages. **b** The graph represents the optical density quantification of the bands for each experimental group (*p < 0.003 for differences between P13 and P28 in control and rd10 mice)

We also aimed to analyze whether a specific subtype of the macroautophagy pathway, CMA, was affected by RP or by retinal development. For this, we used a specific antibody that recognizes the splicing form of lysosome-associated membrane protein (LAMP) called LAMP-2A. Western blotting analysis showed an increase in LAMP-2A expression during postnatal retinal development (with a statistically significant difference between P13 and P28 *p* < 0.003, Fig. 7). No significant difference was found in the LAMP-2A levels between the control and rd10 mice. However, although either the control and rd10 mice showed similar levels of LAMP-2A at P13, the increase in LAMP-2A is more evident in the rd10 retinas between P21 and P28. Retinal LAMP-2A immunohistochemistry was also performed and we demonstrate that this protein is mainly expressed in RPE (Supplementary Figure 2).

## Discussion

### Characterization of the cell death timeline during retinal development in the control and rd10 mice

Several animal models have been proposed for RP research over the years. However, there are variations in the degeneration timelines and acuteness. In some models, degeneration occurs at the same time that the retina is developing. Retinal degeneration in the rd10 mouse is caused by a point mutation on exon 13 in the *Pde6b* gene^40–42^. We studied the time course of retinal cell death in rd10 mice compared to that of the controls. The retina is not completely developed at P5, and cell death was observed in different retinal layers in both the control and rd10 mice, corresponding to the normal apoptosis process that occurs during retinal development (Fig. 2). However, between P13 and P21, only rd10 mice showed a significant reduction in the number of photoreceptors in the retina (Fig. 1). This reduction was accompanied by an increase in TUNEL-positive cells (Fig. 1). At P28, there was almost no cell death in the control retinas, while some apoptotic events still occurred in the rd10 mice. These results are in agreement with previous reports^30^.

We also analyzed markers of oxidative stress and autophagy in the retinas of control and rd10 mice at P13, P21, and P28, to ensure that our results were not influenced by the partial development of the ONL. This comparison also allowed us to observe how the ONL changed during the retinal degeneration of the rd10 mice.

### Characterization of the time course changes in retinal thiol metabolism of the control and rd10

Our group has already reported changes in the retinal GSH and GSSG concentrations in control and rd1 (another RP animal model) retinas with age. In that study, we reported changes in GSSG in rd10 retinas, but only P21 rd10 mice were studied^43^. In the present study, we describe changes in retinal GSH, GSSG, and glutamate with age in rd10 mice and their corresponding control mice.

In our study, retinal GSH concentrations decreased in the rd10 mice at P28 compared to the control mice. This decrease may indicate that the retina in the rd10 mice is less prepared to deal with additional sources of pro-oxidants, as previously described^43^.

Regarding GSSG concentration and GSH/GSSG ratio, there were no differences between the retinas of the rd10 and control mice. However, an increase in GSSG retinal concentration and a decrease in the GSH/GSSG ratio were observed in both the control and rd10 mice at P21 and P28 when compared to P13. These results suggest that there is an increase in retinal oxidative stress as the animal's age increases, as well as after their eyes are opened and exposed to light, inducing an increase in oxidative reactions and in the production of several reactive oxygen species. Different explanations can be suggested for the changes in GSSG concentration or GSH/GSSG ratios that are not accompanied by any change in GSH concentration: in mammalian cells, three mechanisms serve to maintain the GSH homeostasis, de novo synthesis, the catalyzed reduction of GSSG, but also an extracellular uptake (retinal GSH may be increased because there is an uptake from other parts of the visual system). It can be suggested that though GSSG is not being reduced to GSH in our animal retinas, GSH concentration does not change because it may be synthesized by other means.

In addition, GSSG concentrations and GSH/GSSG ratios do not change after 4 h of darkness (Supplementary Figure 1), and it could be concluded that the increases observed with age in GSSG are not due to diurnal variation with lighting conditions and can be related to the increase in light intensity that occurs when mice open their eyes.

We did not observe any difference in the retinal glutamate concentrations in control and rd1 mice. In addition, no changes in retinal glutamate concentrations were detected with age in the control and rd10 mice. Glutamate is the main excitatory neurotransmitter in the vertebrate retina^44,45^. Other studies have demonstrated that retinal glutamate content increases during the postnatal period with the generation and maturation of glutamatergic cells^45^. However, these cells are formed before birth and during the first week after birth. We did not find any changes in retinal glutamate concentrations after P13, in accordance with other results that have demonstrated that retinal amino acid levels stabilize by the time of eye-opening^46^.

### Changes in other markers of oxidative stress markers

Herein, we demonstrate an increase in oxidatively damaged DNA in rd10 retinas compared to controls (Fig. 3e, f). Lipid peroxidation markers (4-HNE staining) increased in the control retinas with age and after mice opened their eyes and began to receive light. However, in rd10 retinas the intensity of 4-HNE was increased earlier than in control retinas. These results lead us to confirm that there is some kind of oxidative stress associated with the light exposure after mice opened their eyes, but that the changes in oxidative stress are more important in rd10 retinas than in controls.

### Characterization of the time course changes in the retinal expression of autophagy markers in control and rd10 mice

Recent findings have shed light on the important role of autophagy in the maintenance of photoreceptor homeostasis and preserving visual function^47,48^. We investigated how macroautophagy and CMA varied during retinal development and during the disease course in the rd10 mice. This study analyzes changes in several autophagy markers, including LC3 II/I ratio, Beclin, Atg 5, and Atg 7.

LC3-II protein is a hallmark of autophagosome formation. LC3-I is transformed into a lipidated LC3-II, which can be detected by western blot^23^. Beclin also plays a key role in autophagy. By acting as a core subunit of the phosphatidylinositol 3-phosphate (PI3K) complex, Beclin mediates the formation of PI3K and is involved in the initiation of autophagosomes. Beclin may also coordinate and regulate autophagy and membrane trafficking. Atg5 and Atg7 are involved in autophagic vesicle formation. Autophagosome elongation and closure involves an Atg7-dependent Atg12–Atg5 conjugation system that controls the lipidation, by phosphatidyethanolamine (PE), of LC3.

In our study, we did not find any statistical significant differences in the retinal levels of LC3, Beclin, Atg5, and Atg7 between control and rd10 mice, but there was a slight increase in the LC3 II-I ratio with developmental age (Fig. 4) in both the control and rd10 mice. Other studies have shown that there is a reduction in the lipidated form of LC3 (LC3-II)^49^ and that an autophagy blockade occurs before the cell death peak in rd10 mouse retina. While LC3 levels serve as a readout of autophagosome number at specific times, they do not offer any important information about autophagy per se, because autophagosome levels can also increase if autophagy is blocked in the last phases of the process. Further studies are needed to study the autophagic flux through the entire process^50^.

CMA is a form of selective autophagy that, to date, has only been described in mammals^51^. The lysosomal membrane protein LAMP-2A, which functions as a lysosomal receptor, participates in the translocation of unfolded polypeptides across the lysosomal membrane^51^.

CMA upregulation has been previously described in vitro in macroautophagy-deficient lines^52^, and CMA activity increases with the age^53^. Previous studies have demonstrated that Atg5 deficiency in neuronal precursors induces photoreceptor cell death. It has been suggested that this partial loss of Atg5 triggers a compensatory upregulation in CMA, an effect that is also observed in the retina^54^. Retinal function defects have been detected following Atg5 deletion in rods and cones^55,56^.

Our study demonstrates for the first time that there is an increase in retinal LAMP-2A concentrations in both the control and rd10 mice at P28 when compared to P13 (after mice opened their eyes). At P28, cell death related to normal retinal development has almost finished.

Although we cannot provide direct evidence about the relationship between CMA and light exposure, some results from other groups can support that speculation: (i) It has been identified a circadian rhythm related to variations in photoreceptor autophagy;^57,58^ (ii) enhanced autophagic events have been documented under light exposure conditions;^57^ (iii) in vitro experimentations with cultured retinal cells demonstrated a dynamic change of autophagic events that is inversely correlated with the dose-dependent cytotoxicity of a visual chromophore associated with light-induced retinal toxicity^59^.

Regarding the retinal CMA levels in the rd10 mice, no statistically significant differences in LAMP-2A levels were found compared to the control mice; therefore, we cannot confirm that any alteration of the CMA process occurs in RP. However, although either rd10 and control mice showed similar levels of LAMP-2A at P13, the increase in LAMP-2A is more evident in rd10 retinas between day 21 and 28, suggesting that chaperone-mediated autophagy is somehow compensating the impairment of other proteolytic pathways during retinal degeneration and/or aging in these mice.

Since we found an accumulation of LAMP-2A in rd10 mice, we performed an immunohistochemistry in mice retina and observed that LAMP-2A is expressed in the retinal pigment epithelium (RPE) (Supplementary Figure 2). Increases in LAMP-2A retina over time do not seem to be influenced by the reduction in the number of photoreceptor cells in the rd10 mice. Our results indicate that the cells in which changes are occurring over time are not photoreceptors; instead, LAMP-2A is mostly expressed in the RPE. In a similar way, we have previously demonstrated that GSH can be found mainly in Müller cells^43^ and HNE that is also increased in rd10 mice retinas is expressed over all retinal cells.

In summary, our results demonstrate that although only slight differences were found in oxidative stress and autophagy markers between the control and rd10 mice in the postnatal days analyzed in this study, there were increases in the GSSG and LAMP-2A concentrations with age, mainly after the mice opened their eyes. This increase in oxidative stress and CMA may indicate a retinal response to the light exposure and should be taken into consideration when studying retinal pathologies.

## Materials and methods

### Animal procedures

C57BL/6 J (control) mice and the rd10 mouse model of retinal degeneration (*Pde6b*^*rd10/rd10*^) were used for this study (mice were a gift from Dr. Hernández from Miguel Hernández University; C57BL/6 J and rd10 mice were congenic). The rd10 mouse model has been described as an appropriate model for retinal degeneration studies. Mutations occurring in rd10 mice are located in the exon 13 of the *Pde6b* gene encoding the phosphodiesterase beta subunit of rods. Mutations affecting phosphodiesterase 6 are the cause of 9–10% of human RP cases, making this animal model a relevant tool to study the cellular and molecular events that lead to photoreceptor death^60,61^.

Animal care and protocols were approved by the Animal Ethics committee of the Universidad CEU Cardenal Herrera and conformed to the ARVO Statement for the Use of Animals in Ophthalmic and Vision Research as well as the Spanish laws regulating animal experiments. The mice were held in cages under controlled conditions of light/dark cycles, temperature and humidity. Animals had free access to water and food. Both male and female mice were used for this study and at least three animals per group were used for each experiment. Retinas were always dissected at the same time of the day (with the exception of the retinas used for determination of GSH concentrations during the night). The retinas for the characterization studies were extracted at P5, P13, P21, and P28. For evaluating the presence of autophagy and oxidative stress, mice were euthanized at P13, P21, and P28. At the indicated days, eyes and fresh retinas were collected. Fresh retinas were rapidly frozen at −80 °C until their manipulation, and eyes were fixed for 2 h with 4% PFA in 0.1 M phosphate buffer, and cryoprotected using increasing concentrations of sucrose up to 30%. The eyes were cut into 10 µm cross sections on a cryostat (Leica CM 1850 UV, Barcelona, Spain) collected on slides and stored at −4 °C until they were used.

### Immunostaining in retinal sections

Sections were rehydrated in PBS and the TUNEL assay was performed with an in situ cell death detection kit (Roche Diagnostics, Mannheim, Germany) according to the manufacturer’s instructions. Fluorescence microscopy was performed using a Nikon DS-Fi1 camera attached to a Leica DM2000 microscope. Sample photographs were taken and analyzed with Leica application and Suite version 2.7.0 R1 software (Leica Microsystem).

Avidin, 4-hydroxynonenal (4-HNE), and LAMP-2A were analyzed by immunohistochemistry. Quantification of immunochemistry was performed on digital images using ImageJ Software. For avidin quantification the number of positive cells was divided by the retinal area. Quantification of 4-HNE was performed by measuring the average staining intensity per pixel in three sections from each sample at a similar distance from the optic nerve and this result was also divided by the retinal area. Two retinal areas were considered, the whole retina or only the outer segments of the photoreceptors.

### Biochemical assays

Two retinas of the same mice were homogenized in 200 μl of phosphate buffer. Protein content was measured using the Lowry method^62^. Retina concentrations of GSH, GSSG, and glutamate were determined using the high-performance liquid chromatography (HPLC) procedure described by Reed^63^.

### Western blot

Pools of two retinas were homogenized manually with RIPA buffer (containing 150 mM of NaCl, 1% NP 40, 0.5% of Na deoxycholate, 0.1% of SDS (sodium dodecyl sulfate) and 50 mM of Tris at pH 8). Protein samples (75 μg) were resolved on 10–15% acrylamide:bisacrylamide gels. The proteins were transferred to nitrocellulose membranes (GE Healthcare Life Sciences, Barcelona, Spain), and blocked for 1 h with 0.01 M PBS-Tween 20 0.1% with 5% w/v non-fat milk. Membranes were then probed with the following antibodies: LC3, Atg7 (both from Cell Signaling, MA, USA), Beclin (Santa Cruz, Santa Cruz, USA), LAMP-2A (Invitrogen, CA, USA), and Atg5 (Sigma-Aldrich, Spain). The membranes were incubated for 1 h at room temperature and the bound antibody was visualized with a horseradish peroxidase-coupled secondary anti-rabbit (F(ab')2 – HRP, goat anti-rabbit) (Santa Cruz Biotechnology, Santa Cruz, USA). The signal was identified with an enhanced chemiluminescence (ECL) developing kit (Amersham Biosciences, Buckinghamshire, UK) and blots were measured by densitometry with the help of the ImageQuant™TL (GE Healthcare Life Sciences, Barcelona, Spain).

Table 1 shows the information about the antibodies used in this study in the immunohistochemistry and western blot techniques.

**Table 1:** List of antibodies used in this study

Antibody name	Source	Manufacturer	Catalogue number
Anti-Atg5	Rabbit polyclonal	Sigma-Aldrich (Merck)	ABC14
Anti-Atg7	Rabbit polyclonal	Cell Signalling Technology	2631S
Anti-BECN1	Rabbit polyclonal	Santa Cruz Biotechnology	sc-11427
Anti-L2A	Rabbit polyclonal	Invitrogen	51–2200
Anti-LC3B	Rabbit polyclonal	Cell Signalling Technology	2775S
Anti-HNE2	Rabbit polyclonal	Alpha Diagnostic	HNE11-S

### Statistical analysis

The results are presented as mean values ± standard deviations from at least three animals in each group. Variance homogeneity was assessed using Levene’s Test of Homogeneity of Variance. A two-way analysis of variance (ANOVA) was applied (age was the primary treatment and strain was selected as the second factor). When the ANOVA indicated a significant difference, we applied a post hoc Dunnett’s T3 test. The SPSS software package version 16.0 was used, and the level of significance was set at *p* < 0.05.

## Electronic supplementary material

Supplementary Figure 1

Supplementary Figure 2

Supplementary figure legends
