# Supplementary figures and images for: Oxidative stress and autophagy-related changes during retinal degeneration and development
Source: Cell Death Dis. 2018 Jul 24;9(8):812. doi: 10.1038/s41419-018-0855-8 (PMC6057918; doi:10.1038/s41419-018-0855-8)

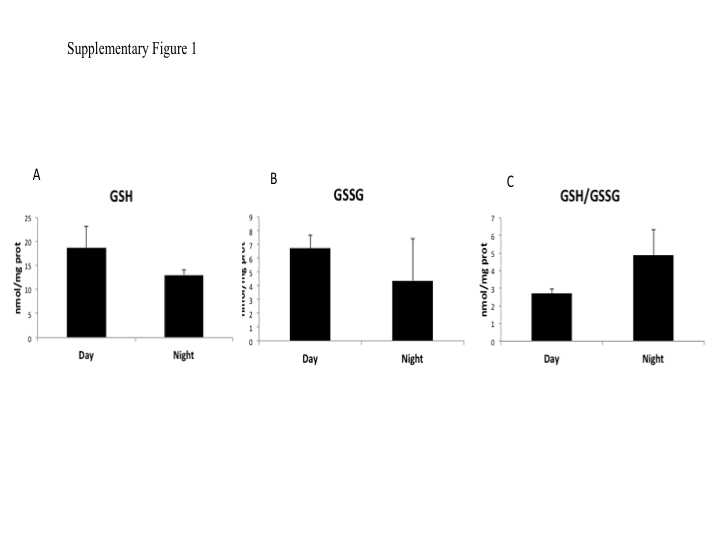

Supplement: Supplementary file 1 — Supplementary Figure 1 [file 41419_2018_855_MOESM1_ESM.tif]

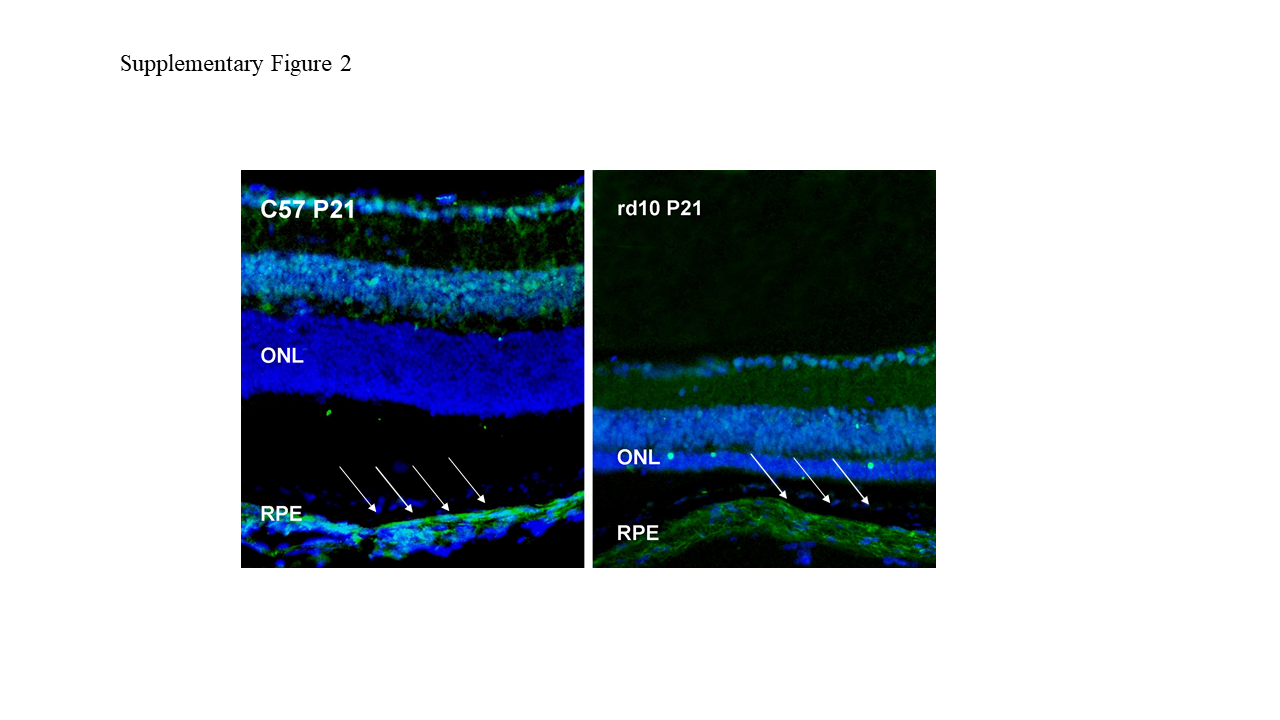

Supplement: Supplementary file 2 — Supplementary Figure 2 [file 41419_2018_855_MOESM2_ESM.tif]
