# Supplementary material for: Oxidative stress and autophagy-related changes during retinal degeneration and development
Source: Cell Death Dis. 2018 Jul 24;9(8):812. doi: 10.1038/s41419-018-0855-8 (PMC6057918; doi:10.1038/s41419-018-0855-8)
Supplement: Supplementary file 3 — Supplementary figure legends [file 41419_2018_855_MOESM3_ESM.docx]

**Supplementary figure legends**

Supplementary figure 1. GSH retinal concentrations do not vary with lighting conditions in rd10 mice. (A) Mean retinal levels of GSH in control mice at P21 during day and night. (B) Mean retinal levels of GSSG in control mice at P21 during day and night. (C) The retinal GSH:GSSG ratio in the P21 control mice retina during day and night.

Supplementary figure 2. LAMP-2A expression in retina of control and rd10 mice. Representative images of LAMP-2A staining in different retinal sections. Left: control mice; right: rd10 mice
